# The innovation and practice of “Hand as Foot teaching method” in the teaching of motion system injury course

**DOI:** 10.1186/s12909-021-02944-w

**Published:** 2021-10-30

**Authors:** Bin He, Qiang Li, Jianmin Zhao, Rui Liu, Yizhou Li, Yafei Xu

**Affiliations:** 1 Inner Mongolia Medical University, First Clinical Medical College, Hohhot North Street, Inner Mongolia, 010050 China; 2 Inner Mongolia Medical University, Department of Orthopedics, Affiliated Hospital of Inner Mongolia Medical University, Hohhot North Street, Inner Mongolia, 010050 China

**Keywords:** Hand as Foot teaching method, Motion System Injury Course, Medical education, Innovation

## Abstract

**Background:**

In view of the teaching characteristics of the motion system injury course and the actual clinical teaching. The orthopedic teaching team of the Affiliated Hospital of Inner Mongolia Medical University took the lead in proposing the "Hand as Foot teaching method" and applied it in clinical teaching. Through this teaching method, students’ understanding and memorization of key and difficult issues in motion system injuries are strengthened, teacher-student interaction is increased, and teaching effect is improved.

**Methods:**

The "Hand as Foot teaching method" was used to teach the key and difficult problems to the clinical undergraduate medical students of Inner Mongolia Medical University, and the teaching process was complemented by PPT + model teaching aids.

**Results:**

The "Hand as Foot teaching method" is generally welcomed by medical students and has achieved good teacher-student interaction, and is effective in understanding and remembering difficult knowledge points.

**Conclusion:**

The "Hand as Foot teaching method" is a novel teaching method that can be applied in clinical teaching. This image teaching method improves the teaching effect, enlivens the classroom atmosphere, and enhances the interaction between teachers and students, which makes students’ learning process from abstract to intuitive, from simple rote memorization to comprehension and memory, and achieves satisfactory results. It can complement each other with the traditional teaching method of pure PPT + teaching aids model, and to some extent it is worth promoting in the motion system injury courses.

The teaching objectives of the Motion System Injury Course are to familiarize students with the basic theories of orthopedic surgery, master the clinical theoretical knowledge and clinical skills of common and multiple diseases of orthopedic surgery, and apply them to clinical practice. The motion system course includes thirteen chapters, 69 sections, and 32 h of class time. At present, the problems of teaching in our school are mainly in the following three aspects: (1) the content is large, the number of hours is less, it is difficult for teachers to speak comprehensively and thoroughly; (2) this course is closely related to anatomy and biomechanics, which requires students to have a good sense of space and logical thinking ability, especially the occurrence of fracture sites, typology, symptoms and typical signs are not only closely related to anatomy, but also often need abstract and difficult to understand the mechanism to interpret. Therefore, this part is difficult to master and memorize, and may even cause confusion; (3) The main teaching means used in the course is PPT(“PPT” is used through the text to designate powerpoint presentations) + model teaching aids. Although the introduction of multimedia in recent years has greatly enriched the teaching methods [1], but multimedia + pictures + model teaching aids still have many shortcomings in teaching, multimedia, PPT mainly shows two-dimensional pictures, presenting students with a flat picture, while the real skeleton is a three-dimensional structure, so multimedia can not give students a real feeling. In addition, due to the limited space, students cannot observe the demonstration of teaching aids at a close distance, or the teaching of models is limited to the classroom, so students cannot review them after class, which cannot improve the quality of teaching [2]. VR technology was formally introduced in 1989 by Jaron Lanier, an American. This technology simulates human vision, hearing, touch, etc. Using computer graphics technology, simulation technology and display technology to achieve real-time interaction between people and machines, so that users “immersive” [3]. In recent years, with the maturity of VR technology, it have be widely used in teaching, and achieved good teaching results [4]. China is a developing country, especially the economic underdevelopment in the central and western regions of China, and many advanced technologies are difficult to spread, and the level of medical education is relatively backward. Because of the single and boring teaching means, low investment in teaching funds, some advanced teaching means are difficult to use, so it is difficult to show the teaching content vividly and graphically, and it is difficult for students to understand and remember some key and difficult problems. Therefore, how to make these problems clear and thorough, so that students can more easily understand and remember, is a major problem in our theory class teaching. In view of the teaching characteristics of the movement system course and the actual clinical teaching, the orthopedic teaching team of the Affiliated Hospital of Inner Mongolia Medical University took the lead in proposing the “Hand as Foot teaching method” and applied it in the clinical teaching, which has achieved good results.

## The innovative source of “Hand as Foot teaching method”

The “Hand as Foot teaching method” is inspired by the theory of evolution, which states that humans evolved from reptiles, from horizontal movement of the limbs to vertical movement of the lower limbs. Although the upper and lower extremities of human beings are different in form, there are many similarities in anatomy, movement, function and biomechanics between the upper and lower extremities. On the one hand, we can take advantage of the high similarity of anatomical structures such as bones, nerves, and blood vessels in the upper and lower extremities, and then demonstrate the lower extremities with the upper extremities to build a three-dimensional understanding of anatomy, and in addition, we can use this method to perform dynamic simulations to understand mechanism problems [5]; on the other hand, because of the high structural similarity between the upper and lower extremities, upper and lower extremity fractures are also highly similar in etiology, clinical manifestations, and treatment. We can guide students to find the “similarities and differences” between upper extremity diseases and lower extremity diseases, and then compare and summarize the knowledge, which can deepen understanding and enhance memory, while teachers can integrate and optimize the teaching content of upper and lower extremity injuries and diseases, which can reduce the teaching time and improve teaching efficiency.

## Application of “Hand as Foot teaching method” in the teaching of motion system

For the clinical undergraduate medical students of Inner Mongolia Medical University, the “Hand as Foot teaching method” combined with PPT + model teaching aids was used to teach the motion system course in a complementary way. For the characteristics of the motor system course, we segmented the knowledge points: anatomical knowledge about injury, fracture classification, fracture displacement characteristics, fracture deformity and mechanism, fracture injury mechanism and characteristics, clinical manifestations and treatment principles of the disease.

### Demonstrating the lower extremity in teaching through the upper extremity

Demonstration of lower extremity injury through the upper extremity the relevant anatomical knowledge、fracture classification、fracture displacement characteristics、fracture deformities and mechanisms of occurrence、fracture injury mechanisms and characteristics can be explained visually.

#### Simulation demonstration of relevant anatomical knowledge and fracture sites

**Fig. 1:**
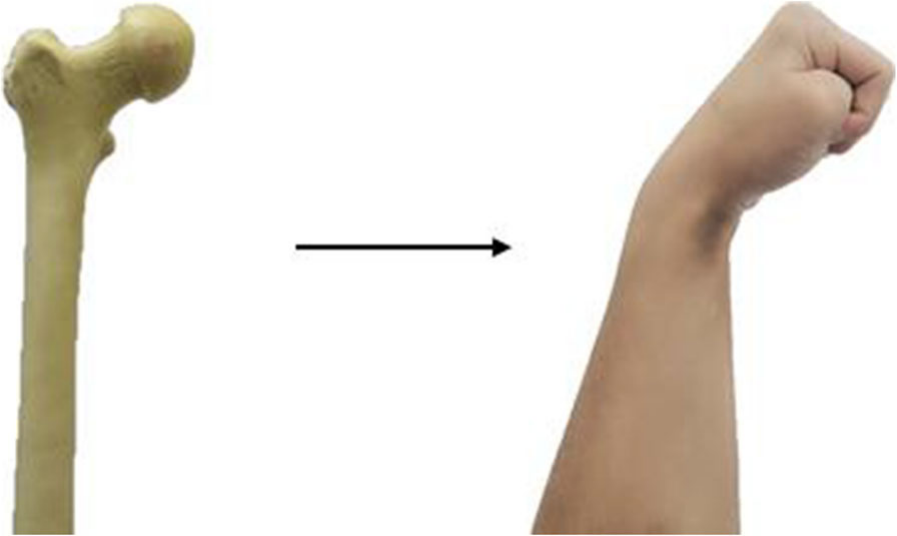
Model building

**Fig. 2:**
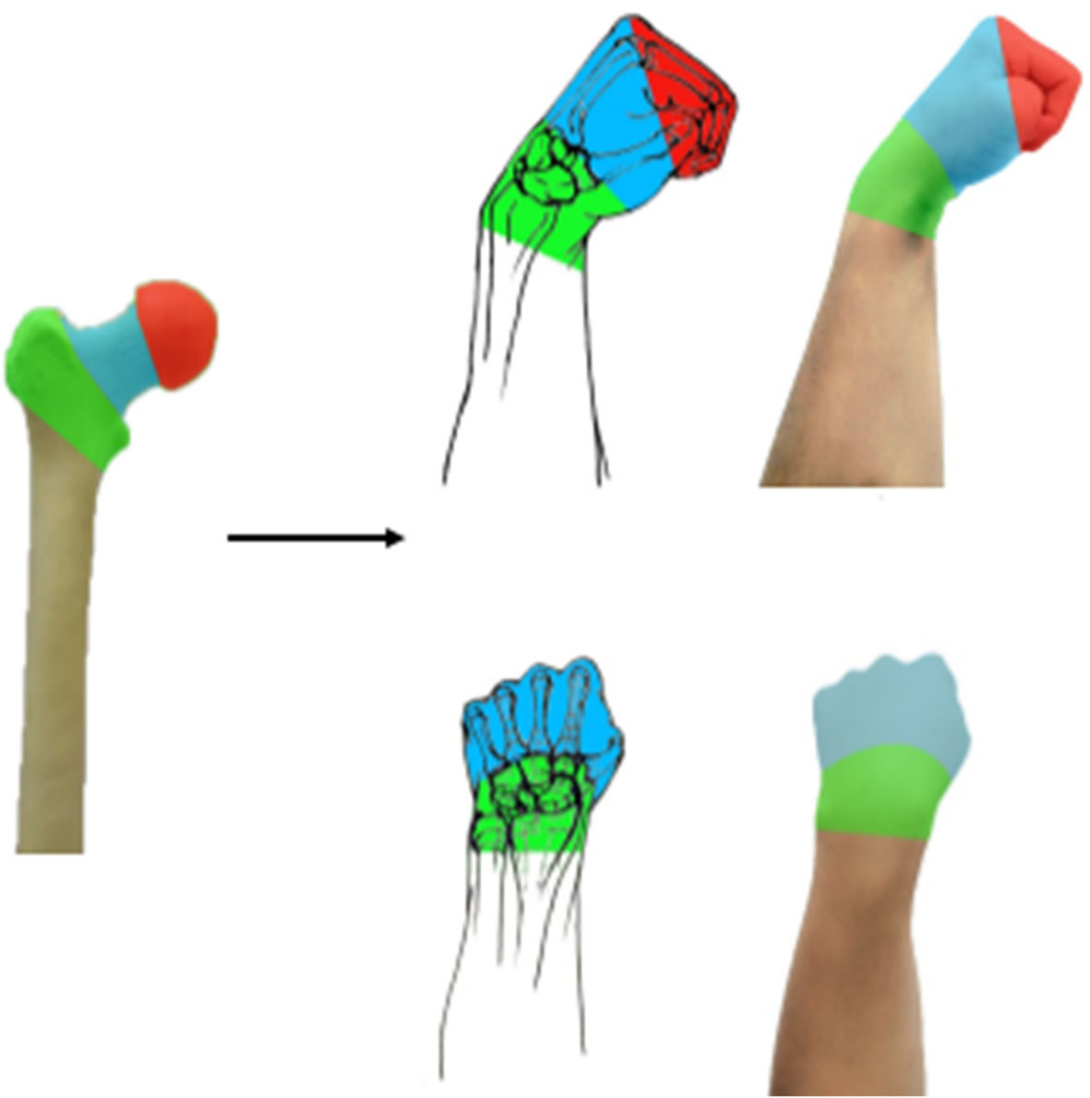
Simulation demonstration of injury-related anatomical knowledge (The red color indicates the femoral head, blue indicates femoral neck, green indicates intertrochanteric femur)

The morphology of the wrist during palmar flexion of the hand is very similar to the anatomy of the proximal femur. In the classroom, the structure of the proximal femur was first simulated by modeling the wrist in the palmar flexion fist position to introduce it into our theory class (Fig. 1). The fist posture represents the femoral head, the metacarpal bone from the metacarpal head to the base of the metacarpal bone represents the femoral neck, the radial tuberosity represents the lesser trochanter, and the base of the metacarpal bone to the lower edge of the radial tuberosity represents the intertrochanter (Fig. 2).

#### Simulation demonstration of fracture classification

**Fig. 3:**
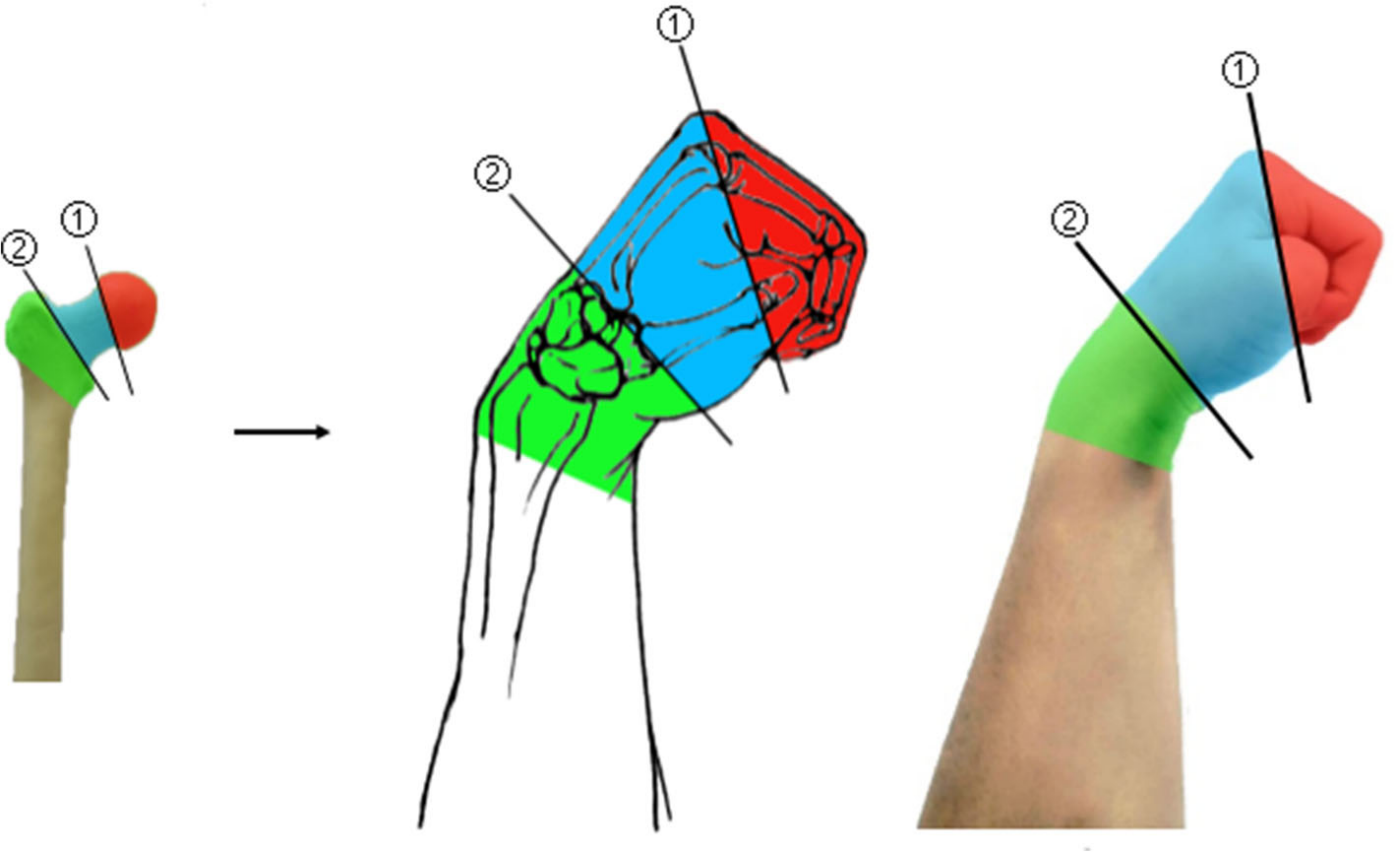
Demonstration of the classification of injury-induced fractures

**Fig. 4:**
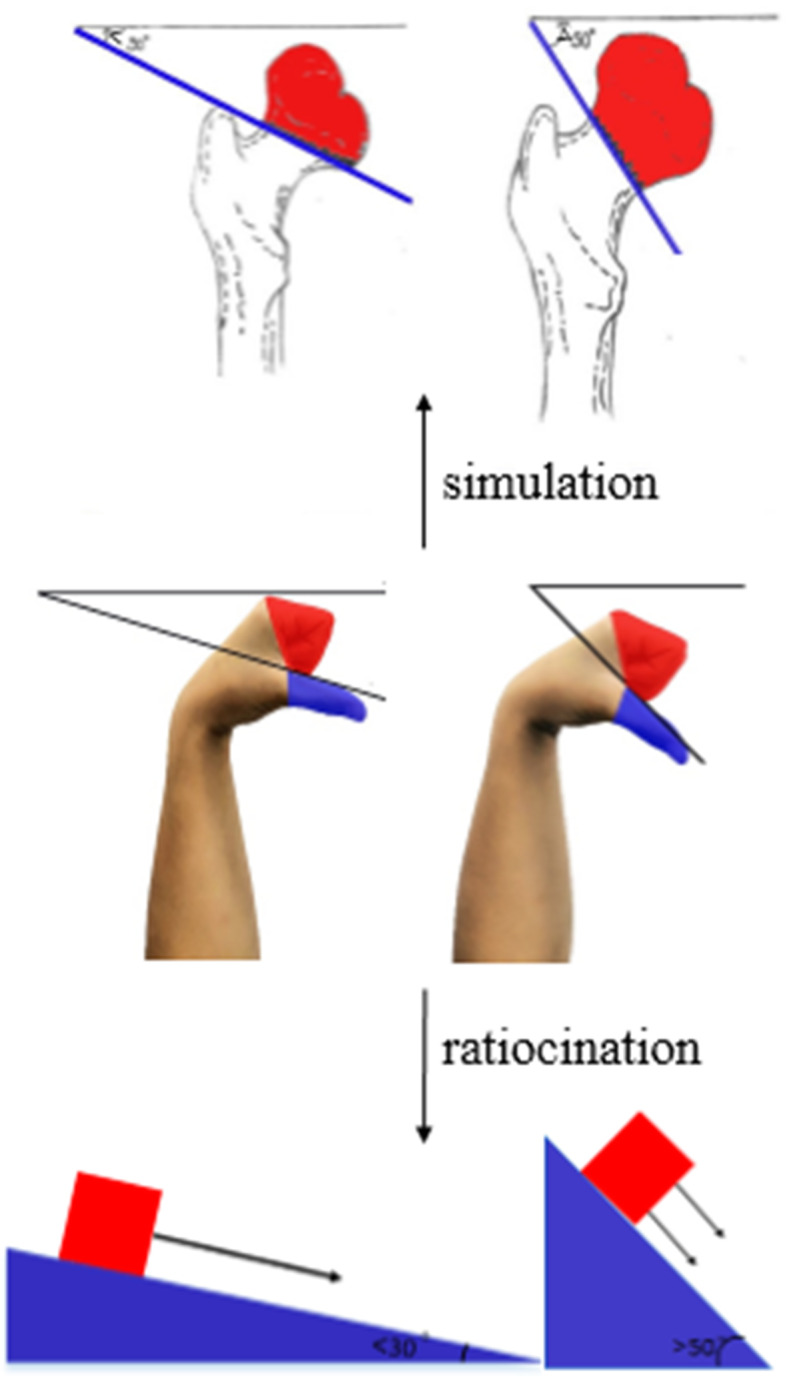
Demonstration of the Pauwels angle and its mechanism

When explaining the fracture classification, the femoral neck fracture is divided into three types of fractures according to the location: subhead type, neck type, and basal type. We simulated the fracture by “palmar flexion of the wrist”, a straight line connecting the 1–5 metacarpal bones was recorde① straight line connecting the 1–5 metacarpal bases was recorded as line②, the line① represented the subcapital fracture, the line② represented the basal fracture, and ①②between the two lines was the cervical fracture (Fig. 3). The Pauwels angle (the angle between the fracture line and the horizontal line) is divided into adductor and abductor fractures, with the adductor fracture having a Pauwels angle < 30º, which is a stable fracture, and the abductor fracture having a Pauwels angle > 50º, which is an unstable fracture. In this model, the fist represents the femoral head and the direction of the thumb represents the direction of the fracture line, so the direction of the thumb and the horizontal line are the Pauwels angle. When explaining the relationship between the Pauwels angle and stability, students are asked to do the “thumb-wrist joint palmar flexion fist potential position” while having their brains build a model in which the thumb is imagined as a plane and the fist potential is imagined as an object located in that plane. The more the plane is tilted, the more likely the object will move along the plane and slide faster, thus inferring the physical property that the plane is stable and the inclined plane is unstable, which leads students to deduce that the more the fracture line is tilted, that is, the larger the Pauwel angle, the more unstable the fracture is (Fig. 4).

#### Simulation demonstration of fracture displacement characteristics

**Fig. 5:**
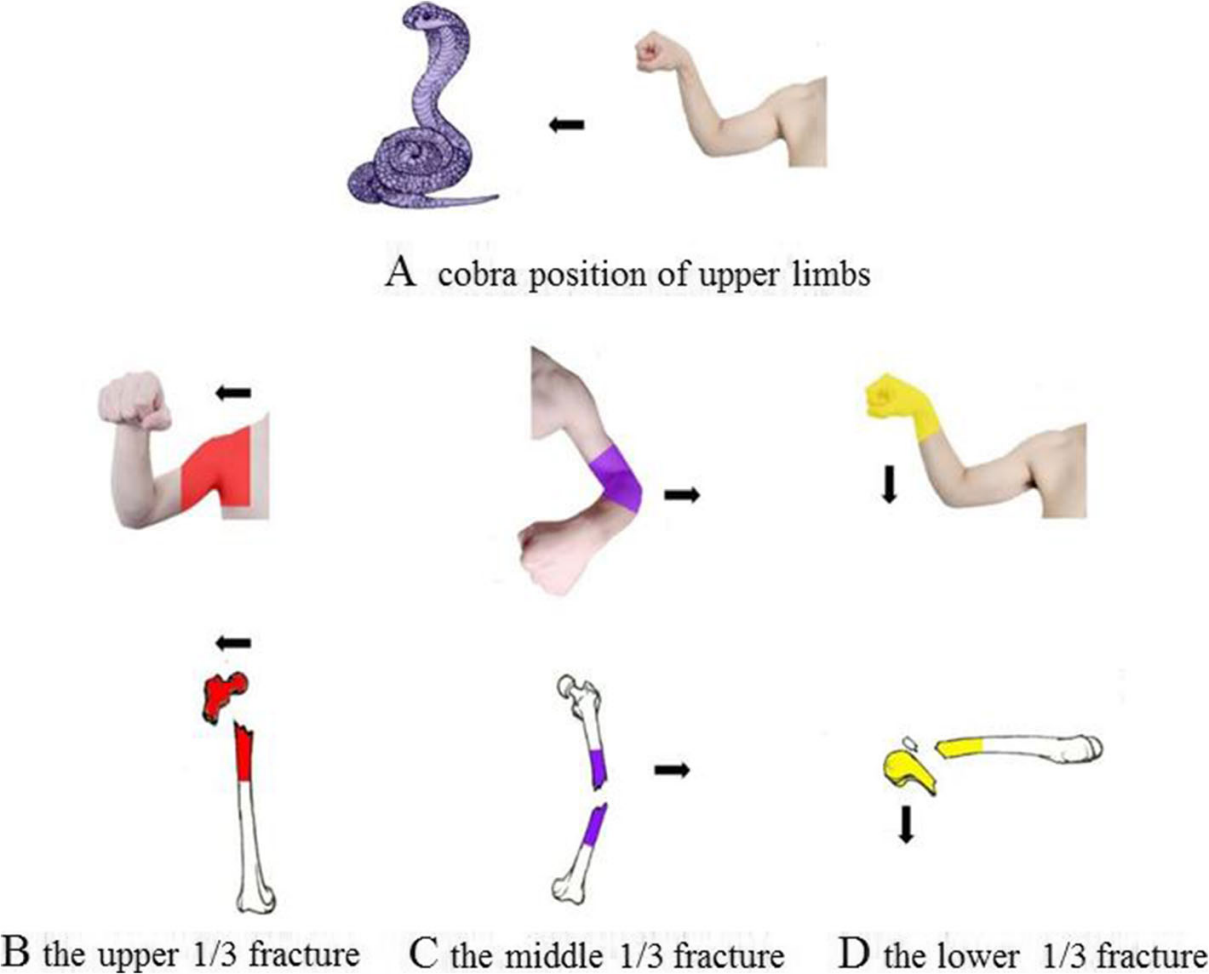
Simulation demonstration of injury-induced fracture deformity

Femoral stem fractures can be divided into upper 1/3, middle 1/3 and lower 1/3 fractures. The typical displacement of each component is due to the traction of the attached muscles. Fracture of the upper 1/3 of the femoral stem: the proximal end of the fracture is displaced in the forward, outward and outward rotational directions due to the pull of the iliopsoas, gluteus medius and gluteus minimus muscles, and also the externally rotating muscle groups. Fracture of the middle 1/3 of the femoral stem: generally speaking, there is an outward angular deformity due to the action of the internal thigh muscles. Fracture of the lower 1/3 of the femoral stem (supracondylar fracture of the femur): the distal fracture end is displaced posteriorly due to the traction of the gastrocnemius muscle and the gravity of the limb. It is not difficult to understand the fracture displacement characteristics by memorizing the starting and ending points of this part of the muscle as well as some biomechanical basis, but it is difficult to memorize. Even students who master this part in the classroom may confuse the three typical directions of displacement or forget them as time goes on. We use the upper limb to simulate the shape of a cobra to describe the three typical displacements of femoral stem fractures, which we call the “upper limb cobra position” image teaching method (Fig. 5). Make the posture of hand clenching, wrist flexion and elbow flexion, and shoulder abduction with one upper limb. At this point, the upper limb looks like an upright cobra (Fig. 5A). Using the shoulder joint to represent the fracture of the upper 1/3 of the femur, the shoulder joint was found to be in flexion, abduction and external rotation (Fig. 5B); using the flexed elbow joint to represent the fracture of the middle 1/3 of the femur, the elbow joint was found to be exactly angled outward (Fig. 5C); using the fist to represent the femoral condyles and the wrist joint to represent the lower 1/3 of the femur (Fig. 5D), the wrist joint of the fist was found to be exactly posterior.

#### Simulation demonstration of fracture deformity and mechanism of occurrence

**Fig. 6:**
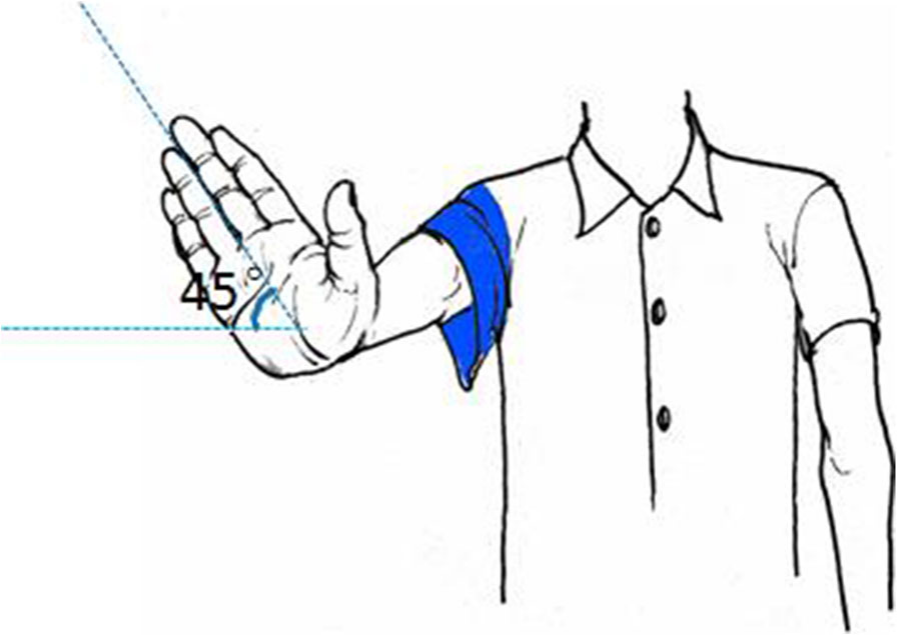
Demonstration of femoral neck external rotation deformity angle

**Fig. 7:**
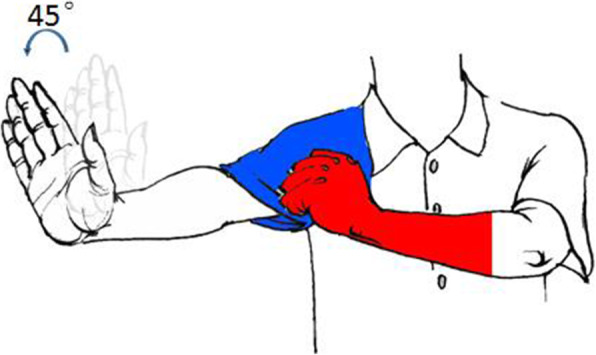
Demonstration of femoral neck external rotation deformity angle mechanism

Extend the forearm and extend the wrist joint dorsally to simulate the shape of the lower extremity, where the “hand” is equivalent to the “foot”, the “wrist joint” is equivalent to the “ankle joint”, the “elbow joint” is equivalent to the “knee joint”, and “shoulder joint” is equivalent to “hip joint”。When we externally rotate the forearm 45°-60°, we make the typical deformity of a femoral neck fracture (Fig. 6). In understanding the deformity angle, the same method is used. We compare the sleeve of the half-sleeve we are wearing to the hip capsule, and the femoral neck fracture occurs inside the sleeve (intracapsular fracture), and when the femoral neck fracture occurs, one hand makes the external rotation deformity, and one hand tugs on the sleeve to simulate the capsule pulling so it cannot be over-externally rotated, and the external rotation angle is 45°-60°. The pulling effect of the joint capsule on the femoral neck is simulated in the left forearm in red (Fig. 7).

#### Simulation demonstration of fracture injury mechanism and characteristics

**Fig. 8:**
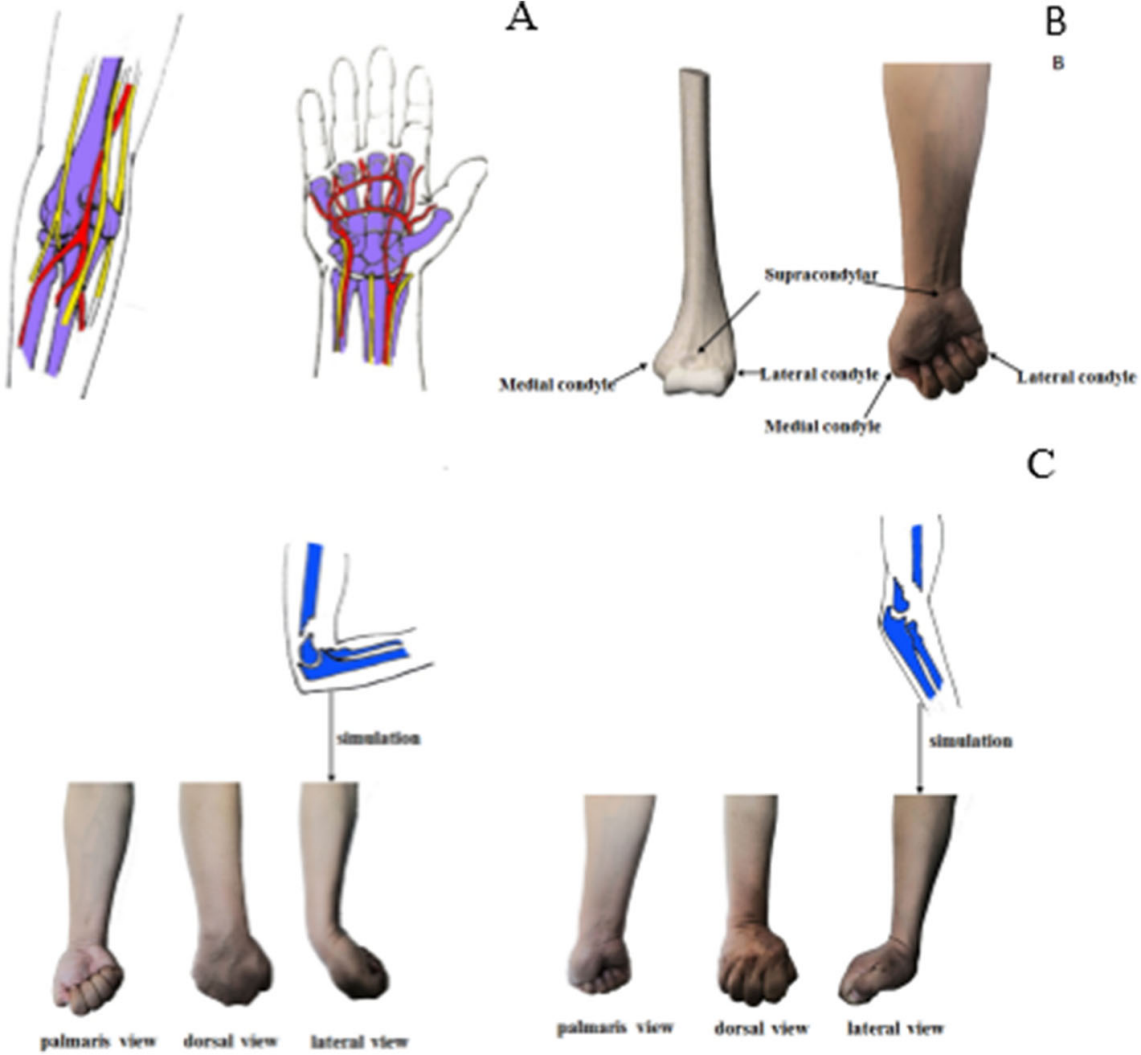
Simulation demonstration of fracture injury mechanism and characteristics

We found that the anatomy of the wrist is very similar to that of the elbow, with important vascular nerves passing on the palmar side of both the elbow and the wrist, and the nerve and vascular courses and distributions of the two places are also very similar, with the brachial artery and median nerve in the middle of the palmar surface of the elbow, the ulnar nerve medially, and the radial nerve laterally; similarly, the median nerve in the middle of the palmar surface of the wrist, the ulnar nerve medially, and the radial nerve laterally, as well as the continuation branches of the brachial artery, the ulnar andradial artery (Fig. 8A). On the dorsal side, the structure is dermatomal. Therefore, we found that the structure of the wrist can completely mimic the structure of the elbow.

We simulated the normal supracondylar structure of the humerus with a clenched fist wrist joint in neutral position (Fig. 8B). There are two types of supracondylar humeral fractures: extension and flexion type. The mechanism of injury in the extension type is that the elbow is in an extension position with the palm of the hand on the ground, and the fracture line travels anteriorly downward to posteriorly upward, which is likely to cause injury to important nerves and blood vessels on the palmar side. In flexion type, the elbow joint is in flexion and the posterior elbow is on the ground, and the fracture line travels from anterior up to posterior down. The fracture line is oblique from the anterior to the posterior. Because there is less soft tissue behind the elbow, the fracture end is sharp and can puncture the skin to form an open fracture. These two types of fractures are difficult for students to understand and remember, and they are often confused. Therefore, we use the “wrist flexion and extension simulation movement” gesture visual teaching method to understand and remember. We used the wrist palmar flexion to simulate the flexion type, and remembered the characteristics of this type of fracture by observing the skin pattern of the wrist joint. We found that the dermatoglyphs appeared on the palmar side and no dermatoglyphs on the dorsal side, so the dorsal side was tense and the collateral injury occurred on the dorsal side, and the fracture end could easily pierce the skin to form an open fracture because of the structural characteristics of the dorsal side of the skin covered bone. Similarly, we simulated the extension type with dorsal wrist extension, and the lateral wrist dermatoglyphs were oriented anteriorly downward to posteriorly upward, which is exactly the direction of the fracture line of the supracondylar humerus in the extension type, and since there is no dermatoglyph on the palmar side, the secondary injury occurred on the palmar side, and it is easy to damage the vascular nerve (Fig. 8C).

### Analogical induction

Because of the high structural similarity between the upper and lower extremities, students can be guided to look for the “similarities in differences and similarities in differences” between upper extremity injuries and lower extremity injuries in clinical manifestations and treatment principles, and then make analogies and generalizations of knowledge, which can deepen understanding and enhance memory. For example, the clinical manifestations of radial nerve injury in the upper limb and common peroneal nerve injury in the lower limb are highly similar, and the clinical manifestations and treatment principles of ulnar radius fracture and tibiofibular fracture are similar. Here the proximal humerus fracture is summarized by analogy with the proximal femur fracture as an example (Table 1).

**Table 1:** Comparison of proximal humerus fractures and proximal femur fractures

	Proximal humerus fracture	Fracture of the proximal femur
Prevalent population	Seniors	Seniors
Etiology and mechanism	Low energy damage The junction of cancellous and dense bone	Low energy damage The junction of cancellous and dense bone
Fractures site	Anatomic neck fracture of the humerus, surgical neck fracture of the humerus	Femoral neck fracture, intertrochanteric fracture
Vascular, nerve than adjacent	Axillary artery, axillary nerve	Femoral artery, femoral nerve
Treatment	Anatomic neck fracture of the humerus: artificial joint replacement Surgical neck fracture of the humerus: internal fixation	Femoral neck fracture: artificial joint replacement Intertrochanteric fracture of the femur: internal fixation
Complications	Ischemic necrosis of the humeral head	Ischemic necrosis of the femoral head

## Discussion

### Interpretation of “Hand as Foot teaching method”

The theoretical basis of the “Hand as Foot teaching method” is based on the high structural similarity between the upper and lower limbs, which embodies two cores: a gesture and a concept, the gesture is “the upper limb simulates the lower limb”, and the concept is “similarities in differences and similarities in differences”. Through the continuous application, exploration and summary of this teaching method, we found that the method is not limited to the simulation and comparison of upper and lower limbs, as long as there are similarities in limb structure, simulation and comparison can be summarized. For example, we can use the wrist joint to simulate the elbow joint, realizing the upper limb simulation of the upper limb, in addition, because the cervical and lumbar spine structure is similar, so cervical spondylosis and lumbar disc herniation in the etiology, clinical manifestations, treatment principles are highly similar and can be compared and memorized [6].

### The advantages of the upper limb simulating the lower limb in the “Hand as Foot teaching method

In the past, when we conducted theory classes related to motion systems injury, over-reliance on multimedia courseware would make students become passive viewers of multimedia courseware content, lacking sufficient time for reflection, lacking questions and discussion of lecture content, and too little teacher-student interaction, which would also make students inattentive for a long time, leading to fatigue and boring learning [7]. Multimedia courseware picture presentation is only a two-dimensional plane display, the traditional teaching aids are too simple, for the knowledge points are not targeted, only through the wall chart, models and other ways to show the knowledge points rigidly, limiting the teacher’s hands and students’ thinking, in addition, the high cost of orthopedic teaching aids, coupled with the number of medical students, teaching time, space are seriously restricted the use of teaching aids, especially the back row of students listening to the class In addition, the cost of orthopedic teaching aids is high. When we teach the anatomical knowledge related to motion injuries, the classification of fractures caused by injuries, the typical signs and deformities, the mechanism and characteristics, etc. Which involve the understanding and recognition of spatial positioning and three-dimensional structure, it is difficult to obtain intuitive understanding by relying on multimedia and teaching aids, which is a clinical teaching point. Through the “Hand as Foot teaching method” demonstration and teaching, not only can guide students to imitate, compared with pictures and anatomical models, more intuitive three-dimensional, it can be said that this gesture image teaching method is a “hand-type teaching aids”. On the one hand, it has the effect of turning abstract into concrete, boring into vivid, and abstruse into plain, which helps students understand and attracts their attention;on the other hand, it enhances the interactivity of the classroom [8], enlivens the classroom atmosphere and makes it easy for teachers to explain.

Teachers use the gesture image teaching method to “teach”, while students simulate the gesture image teaching method to “learn”, the organic combination of “teaching” and “learning” is realized.At the same time, this method of teaching gestures is also a “manual teaching tool”.which not only brings the teacher closer to the students, but also indirectly brings the students closer to the “teaching aids”. In particular.When we explain the anatomical knowledge related to motion injuries, the classification of fractures caused by injuries, typical signs and deformities, mechanisms, characteristics, etc.,compared to the static display of traditional teaching aids, this kind of “hand-shaped teaching aids” demonstration and interaction establishes an interesting learning atmosphere, which not only trains students’ hands-on ability, but also guides students to think about the connection of related knowledge, which really makes students “use their hands and brains” and improves students’ mastery of the knowledge of motion system.

### The advantages of analogical induction in the “Hand as Foot teaching method”

The “Hand as Foot teaching method” is permeated with the concept of “similarities in differences and similarities in differences”, which is mainly reflected in the analogy between upper limb and lower limb injuries, which not only facilitates teachers to sort out and optimize the integration of knowledge points, but also shortens the teaching time. For the students, this method not only helps the teacher to sort out and optimize the integration of knowledge points, but also shortens the teaching time. Through this method, the students’ self-learning ability is honed, and they are guided to generalize by analogy. In the process of learning, students not only master knowledge, but more importantly, master a learning method. Therefore, while students learn through the “Hand as Foot teaching method”, based on their own understanding and speculation, students can also innovate and enrich the gesture teaching method, and generalize more knowledge points by analogy, which has greatly enriched the “Hand as Foot teaching method” and promoted the development of medical teaching, forming a virtuous and dynamic cycle of sustainable development.

### Limitations of the “Hand as Foot teaching method”

At present, this teaching method is recognized by students, but this is only the subjective feedback from students and teachers, which is descriptive analysis, and statistical analysis is still needed to further verify the feasibility and teaching effect of this method, so we have to design relevant experiments to further verify the advantages and feasibility of our teaching method. In addition, the gestural image teaching method may cause psychological problems for some students with physical defects. Therefore, this teaching method is still in the exploration stage and needs to be summarized and improved in the actual teaching, pending more active verification.

## Concluding remarks

Through the “Hand as Foot teaching method”, teachers can combine “teach” and “learn”, students can combine “hand” and “brain”, and teaching methods can combine “multimedia textbooks” and “hand-type teaching aids”. In the learning process, students can see, touch, learn and use, and teacher-student interaction has increased, improving the teaching effect. It can complement the traditional teaching method of PPT mode, and to some extent it is worth promoting in motion system injury courses. Especially in some areas where medical education resources are scarce, this image teaching method not only solves the problem of too simple and insufficient number of traditional teaching aids, but also saves teaching expenses.

## Data Availability

Not applicable.

## Abbreviation

PPT: Powerpoint presentations
