# Correction: The innovation and practice of “hand as foot teaching method” in the teaching of motion system injury course

**DOI:** 10.1186/s12909-022-03818-5

**Published:** 2023-01-09

**Authors:** Bin He, Qiang Li, Jianmin Zhao, Rui Liu, Yizhou Li, Yafei Xu

**Affiliations:** 1 Orthopedic Graduate Student, Affiliated Hospital of Inner Mongolia Medical University, Inner Mongolia Medical University, Hohhot North Street, Inner Mongolia, 010050 China; 2 Department of Orthopedics, Affiliated Hospital of Inner Mongolia Medical University, Inner Mongolia Medical University, Hohhot North Street, Inner Mongolia, 010050 China

**Correction: BMC Medical Education (2021) 21:553**

       10.1186/s12909-021-02944-w.

Following publication of the original article [1], the authors requested to correct the affiliation of author Bin He.

The incorrect affiliation is: Inner Mongolia Medical University, First Clinical Medical College, Hohhot North Street, Inner Mongolia 010050, China.

The correct affiliation is: Inner Mongolia Medical University, Affiliated Hospital of Inner Mongolia Medical University, Hohhot North Street, Inner Mongolia 010050, China.

The original article has been corrected.
